# Investigating cochlear cellular dynamics in neurofibromatosis type 2-associated schwannomatosis: a histopathological study

**DOI:** 10.3389/fneur.2025.1650470

**Published:** 2025-08-15

**Authors:** Reef K. Al-Asad, Drew J. Montigny, Jennifer T. O’Malley, D. Bradley Welling, David H. Jung, Andreas H. Eckhard, Judith S. Kempfle

**Affiliations:** ^1^The Eaton-Peabody Laboratories, The Massachusetts Eye and Ear Department of Otolaryngology - Head and Neck Surgery, Boston, MA, United States; ^2^University of Massachusetts Chan Medical School, Worcester, MA, United States; ^3^Harvard Medical School Department of Otolaryngology - Head and Neck Surgery, Boston, MA, United States; ^4^Department of Otolaryngology, UMass Memorial Medical Center, Worcester, MA, United States

**Keywords:** neurofibromatosis type 2, Schwann cells, macrophages, sensorineural hearing loss, NF2-SWN, spiral ganglion neurons

## Abstract

Sensorineural hearing loss (SNHL) is a hallmark symptom in patients with neurofibromatosis type 2-associated schwannomatosis (NF2-SWN), a genetic condition caused by mutations in the Neurofibromin II gene that encodes the tumor suppressor protein Moesin-Ezrin-Radixin-Like Protein (Merlin; also known as schwannomin). These mutations lead to the development of various tumors, including schwannomas, ependymomas and meningiomas along the vestibular nerve and the cerebellopontine angle. Original theories attributed SNHL in NF2-SWN to the mechanical compression of the vestibulocochlear nerve from the tumor itself, in addition to secretion of toxic tumor byproducts. However, the observation that SNHL can progress independently of tumor size and growth dynamics challenges this view and reveals a critical gap in our understanding of its underlying etiology. To better define cochlear changes associated with hearing loss in NF2-SWN, immunohistochemical cell type markers were used on archival postmortem temporal bone samples from both NF2-SWN patients and healthy controls and quantified the number and cellular density of neural (TUJ1), glial (SOX10), and immune cells (IBA1) within apical, middle, and basal turns of the cochlea. Our findings demonstrated a significant loss of spiral ganglion neurons, a slight increase of Schwann cells, and marked activation of cochlear macrophages in NF2-SWN cases. These findings indicate the contribution of cochlear macrophage-mediated inflammation and Schwann cell dysregulation in the pathophysiology of SNHL in NF2-SWN.

## 1 Introduction

Vestibular schwannomas (VS) are benign Schwann cell-derived tumors that arise on the vestibular portions of the vestibulocochlear nerve in over 90% of cases, frequently leading to sensorineural hearing loss (SNHL) (1–3). Most VS cases are sporadic and unilateral; bilateral VSs are pathognomonic for neurofibromatosis type 2-associated schwannomatosis (NF2-SWN), formerly known as neurofibromatosis type 2 (4). NF2-SWN is a fully penetrant, autosomal dominant condition arising from mutations in the *NF2* gene, which encodes the tumor suppressor protein merlin (moesin-ezrin-radixin-like protein) (5). Mutations in the *NF2* gene often include missense or truncating variants, with truncations in exons 2–13, leading to complete loss of merlin and a worse prognosis (6–10). These mutations disrupt merlin’s role in maintaining cellular architecture and regulating tumor growth, resulting in Schwann cell neoplasia (11).

NF2-SWN typically presents in early adulthood, with up to 95% of patients developing bilateral vestibular schwannomas before the age of 30 (12). Despite arising almost exclusively from the vestibular division of the eighth cranial nerve, vestibular schwannomas cause SNHL in over 95% of patients (2, 3, 13). The progression of SNHL in NF2-SWN is typically gradual but can present with sudden or fluctuating patterns (14). A prevailing theory has attributed SNHL to mechanical compression of the cochlear nerve by the growing tumor (15, 16). However, clinical evidence suggests that hearing loss often progresses independently of tumor size or growth rate (17). For example, patients with small, stable tumors may still experience worsening hearing, highlighting the inadequacy of mechanical compression as the sole explanation for SNHL in NF2 (18, 19).

Alternative mechanisms for SNHL in NF2-SWN have been proposed, including ischemic cochlear damage, direct cochlear invasion by the tumor, and neurotoxic effects of immune factors secreted by the tumor (20–22). Recent evidence implicates neuroimmune interactions and inflammation within the cochlear microenvironment as potential contributors to hearing loss in NF2-SWN (22–26). However, changes in the cochlear non-neuronal cell populations, especially the dynamics of glial and immune cell populations, in the cochlear nerve in NF2-SWN remain poorly defined.

This study aims to systematically characterize the changes to the cellular populations, including spiral ganglion neurons (SGN), Schwann cells and cochlear macrophages, present within the cochleae of NF2-SWN patients to better understand the etiology of SNHL. The cell bodies of SGNs reside within Rosenthal’s canal in the modiolus of the cochlea; their axons project peripherally toward hair cells and centrally to form the auditory nerve. Schwann cells reside interstitially with SGNs in Rosenthal’s canal, and along SGN axons. Cochlear macrophages are present in the normal and injured cochlea as resident cells (27). The etiology of hearing loss in relation to vestibular schwannomas remains unclear, and the study of cellular changes in humans has been limited to post-mortem studies, which has produced evidence of degeneration of several cochlear components, including neuronal loss, particularly in cases with significantly affected speech discrimination (14, 18, 19, 28). Some findings further support a theory of ischemic cochlear damage secondary to vascular recruitment and angiogenesis favoring the schwannoma, diverting blood flow from the cochlea, thus comprising its perfusion (29, 30).

Schwann cells are the predominant glial cell type of the peripheral nervous system. They hold several important roles in the cochlea, including myelination of SGN, which is crucial for both speed and synchrony of signal transmission and therefore, hearing. Additionally, they play an important role in maintaining homeostasis and nerve regeneration after damage (31–36). They are known to proliferate in response to damage and a subset may be able to adopt a proneural phenotype when stimulated (37). Schwann cells in NF2-SWN lose gene expression of *NF2*, leading not only to tumorigenesis and tumor expansion, but also to the loss of their repair capacity (11, 38, 39).

Immune cells, particularly macrophages, are increasingly recognized as key players in cochlear dysfunction (40). Innate macrophages, identified by markers such as Ionized calcium binding adaptor molecule 1 (IBA1), CD163, and CD68, are within the spiral ganglia of human temporal bones of individuals with normal hearing (41). In healthy tissues, these macrophages exhibit a ramified morphology with extensive processes, reflecting a quiescent state (41). This morphology enables them to monitor the microenvironment and maintain cochlear homeostasis. Moreover, they assist proliferating Schwann cells in axonal regeneration after injury by phagocytosing myelin to create an environment permissible for axon growth (31). Macrophage involvement of inflammatory processes also exists within the vestibular schwannomas in the internal auditory canal, where they are called tumor-associated macrophages. This activation is likely driven by pro-inflammatory cytokines and chemokines secreted by the tumor and its associated microenvironment that contributes to angiogenesis, extracellular matrix remodeling, and chemokine-mediated signaling (23, 42).

Hearing loss in NF2-SWN therefore likely results from a complex process involving the interplay of neural, glial, and immune factors within the cochlear microenvironment. Although traditional theories of mechanical compression have dominated the understanding of SNHL in NF2-SWN, emerging evidence highlights the contributions of Schwann cell dysregulation, spiral ganglion neuron loss, and macrophage-mediated inflammation (13, 43). These findings not only provide new insights into the pathophysiology of NF2-related hearing loss but also open avenues for targeted therapeutic interventions aimed at preserving auditory function. In this study, the neural, glial, and immune cell populations in human temporal bone samples from NF2-SWN patients were compared with those from individuals with normal hearing.

## 2 Methods

### 2.1 Case selection

Ethics approval for this study was obtained by the Massachusetts Eye and Ear IRB #2021P000332, and the study was conducted in accordance with all applicable institutional and federal guidelines. Archival human temporal bone sections were retrieved from the Massachusetts Eye and Ear Collection by querying the NIDCD National Temporal Bone, Hearing and Balance Pathology Resource Registry with the key words “vestibular schwannoma.” This search identified 28 cases with unilateral or bilateral VS without additional cochlear or retrocochlear pathology. From these, three hematoxylin and eosin (H&E) stained NF2-SWN cases with sufficient quality to be suitable for immunohistological staining and subsequent cell-level analysis, and for which audiometric and detailed medical record data were available to independently confirm the histopathological diagnosis of VS, were selected (Table 1, Patient 1–3). In addition, nine age-matched control cases without documented history of middle or inner ear disease, and age normal hearing were obtained from the Massachusetts Eye and Ear Collection (Table 1, Patient 4–12).

**Table 1:** Characteristics of selected cases for immunohistochemical analysis.

Patient	Age/sex	Condition	Sample side	Audiogram availability
1	70/M	NF2-SWN	AS/AD	Yes
2	43/F	NF2-SWN	AS/AD	Yes
3	18/M	NF2-SWN	AD	Yes
4	24/M	No ear pathology	AD	Yes
5	92/F	No ear pathology	AD	No
6	70/F	No ear pathology	AD	Yes
7	88/M	No ear pathology	AS	Yes
8	86/F	No ear pathology	AD	No
9	66/F	No ear pathology	AS	No
10	56/F	No ear pathology	AD	No
11	38/F	No ear pathology	AD	No
12	78/M	No ear pathology	AD	No

### 2.2 Temporal bone preparation for immunohistochemical staining

All human temporal bone samples were processed to previously published standard methods by the Otopathology Laboratory at Massachusetts Eye and Ear Infirmary. In brief, samples were fixed in formalin, embedded in celloidin and sectioned at 20 μm thickness. Every 10th section was H&E stained. For immunohistochemical staining, samples were adhered to gelatin-coated slides and celloidin was removed using methods previously described (44). The tissue was then rehydrated, and tissue samples underwent heat and pressure-induced antigen retrieval as previously described (45). PAP pen (Vectashield, RRID: AB_2336789) was applied to the slide to create a hydrophobic barrier. Samples were placed in blocking buffer containing normal horse serum (0.3% Triton, 15% Horse Serum in phosphate buffered solution (PBS)) for 2 h. Then washed in PBS for 10 min. Samples were then treated with primary antibody diluted in buffer containing normal horse serum (0.3% Triton, 10% Horse Serum in PBS) with Rabbit-anti-SOX10 (glial marker, 1:100, Abcam 227,680, RRID: AB_2927464), Mouse-anti-Beta-Tubulin-III (“TUJ1,” neuronal marker, 1:250, Biolegend 801,202, RRID: AB_2313773) and/or Rabbit-anti-IBA1 (macrophage marker,1:100, Invitrogen MA5-27726) overnight in a humidification chamber at 4 °C.

Samples were washed in PBS for 20 min and then moved to secondary antibody solution containing normal horse serum (0.1% Triton, 10% Horse Serum in PBS) with Goat-anti-Rabbit-AlexaFluor647 (1:500, Invitrogen A21244, RRID: AB_2535812) and Donkey-anti-mouse AlexaFluor488 (1:500, Invitrogen A21202, RRID: AB_141607) for 2 hours. Samples were washed for 10 min in PBS. Samples were then moved to tertiary antibody solution (0.1% Triton, 10% Horse Serum in PBS) with Donkey-anti-Goat- Alexa Fluor 647 (1:500, Invitrogen A21447, RRID: AB_141844). Samples were washed for 10 min in PBS then moved to DAPI (nuclear counterstain, 1:250) for 7.5 min followed by 20-min wash in PBS. Slides were mounted with clear DAKO mounting medium and cover slipped (ThermoFisher 24×50 #1 coverslips).

### 2.3 Imaging and quantification of neurons, macrophages and Schwann cells

**Figure 1:**
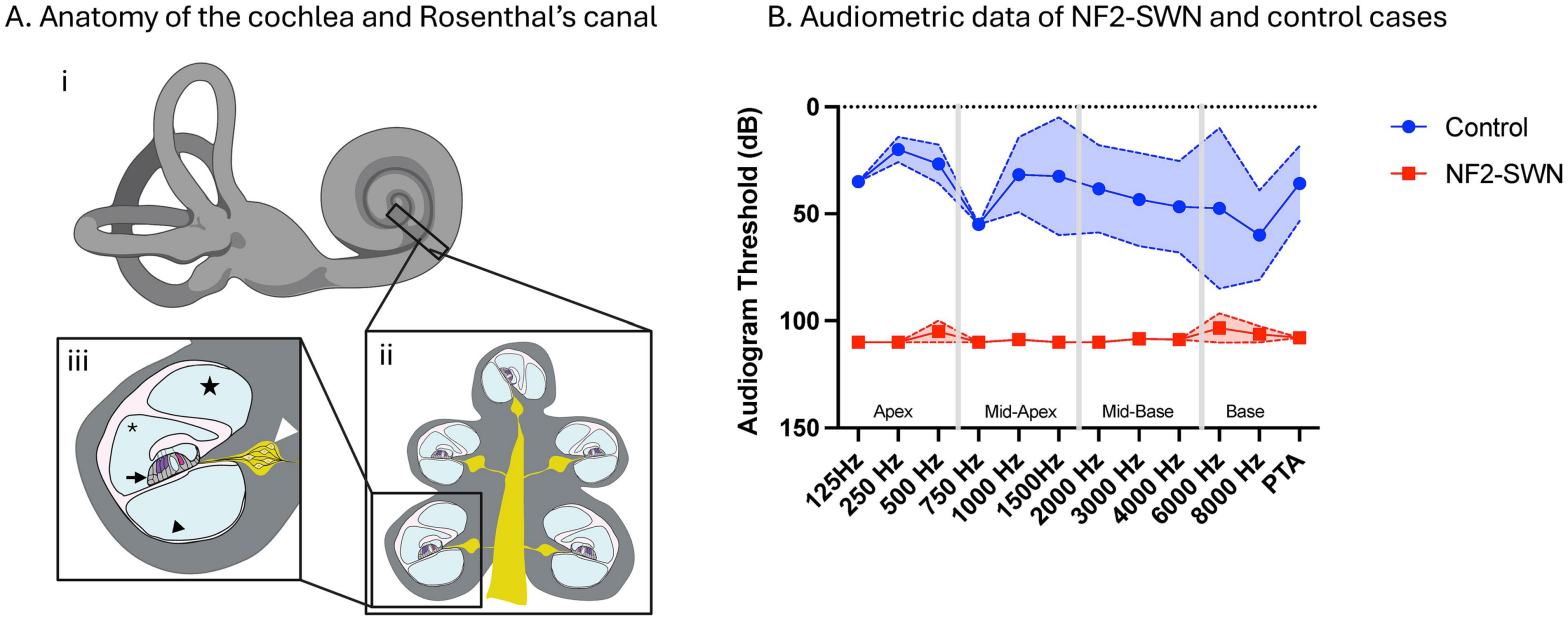
Cochlear anatomy, case selection and audiometric outcomes. **(A) (i)** illustration of the inner ear, including the cochlea and vestibular apparatus. **(ii)** Cross section of a mid-modiolar cochlea section with central axons feeding into the cochlear nerve (yellow). **(iii)** Cross section through one turn of the cochlea, with SGN cell bodies inside Rosenthal’s canal (white arrowhead), organ of Corti (black arrow) containing cochlear hair cells (purple, pink), scala vestibuli (star), scala media (asterisk) and scala tympani (black arrowhead). Created in BioRender. Kempfle, J. (37) https://BioRender.com/gmjutz7. **(B)** Audiometric data with worse hearing across all frequencies in NF2-SWN compared to age matched controls (Control).

Confocal microscopy was performed within 2 days of immunohistochemical staining using Leica SP8 confocal microscope, focused on the cells within Rosenthal’s canal (Figure 1A). Full sample thickness (20 μm) was captured followed by maximum projection. Samples with sufficient tissue preservation and staining quality were included in the analysis. Samples exhibiting poor staining, significant artifact or inadequate cell morphology were excluded from quantitative evaluation. Cells were counted manually in QuPATH for all stains, including TUJ+/DAPI+, SOX10+/DAPI+ and IBA1+/DAPI+. Images were then counted using custom written MATLAB cell counting software for nuclear stains, i.e., SOX10 and DAPI (RRID: SCR_001622). Cell quantification was normalized to cell type per 100 μm^2^.

A custom MATLAB script was developed to perform the macrophage ramification assay. Rosenthal’s canal was manually outlined and fully counted and normalized to 100 μm^2^. Ramification was based on 2D image based on the maximum projection of 20 μm samples. A perimeter and area were calculated for each individual macrophage. Macrophage ramification index was calculated with the equation below for each individual macrophage and subsequently averaged for all macrophages within the spiral ganglia of the image.


(1) $$\text{Ramification index}=2\frac{\sqrt{\text{cell perimeter}}}{\sqrt{\text{cell area}}}$$

Equation 1: Ramification index for macrophage activation.

All statistical analysis was performed using a student’s *T*-test to compare between groups. A two-tailed *t*-test was performed, assuming equal variances. Statistical significance was set at *p* < 0.05 (*), *p* < 0.01 (**), *p* < 0.001 (***), and *p* < 0.0001 (****). Analysis was conducted using GraphPad Prism.

## 3 Results

### 3.1 Audiologic findings

Hearing evaluation using pure tone audiograms identified significantly elevated thresholds, corresponding to worse hearing, across all frequencies in NF2-SWN patients compared to controls. All samples with NF2-SWN demonstrated severe to profound hearing loss (Figure 1B). In our analysis of H&E-stained sections, neuronal and non-neuronal cells within the spiral ganglia could be identified and tissue quality was found to be sufficient for fluorescent immunohistochemistry.

### 3.2 Immunohistochemical findings

To determine the changes to cellular populations within the cochlea in patients with NF2-SWN, immunohistochemistry was utilized to quantify neurons, Schwann cells and macrophages as described above. Confocal images that were captured in each cochlear turn of both NF2-SWN and normal hearing controls were normalized to 100 μm^2^. The sample size of each condition corresponds to the number of images analyzed and varied by the total area of measurement available, in addition to the quality of the tissue sample and staining. Significant changes were present across all cell types.

**Figure 2:**
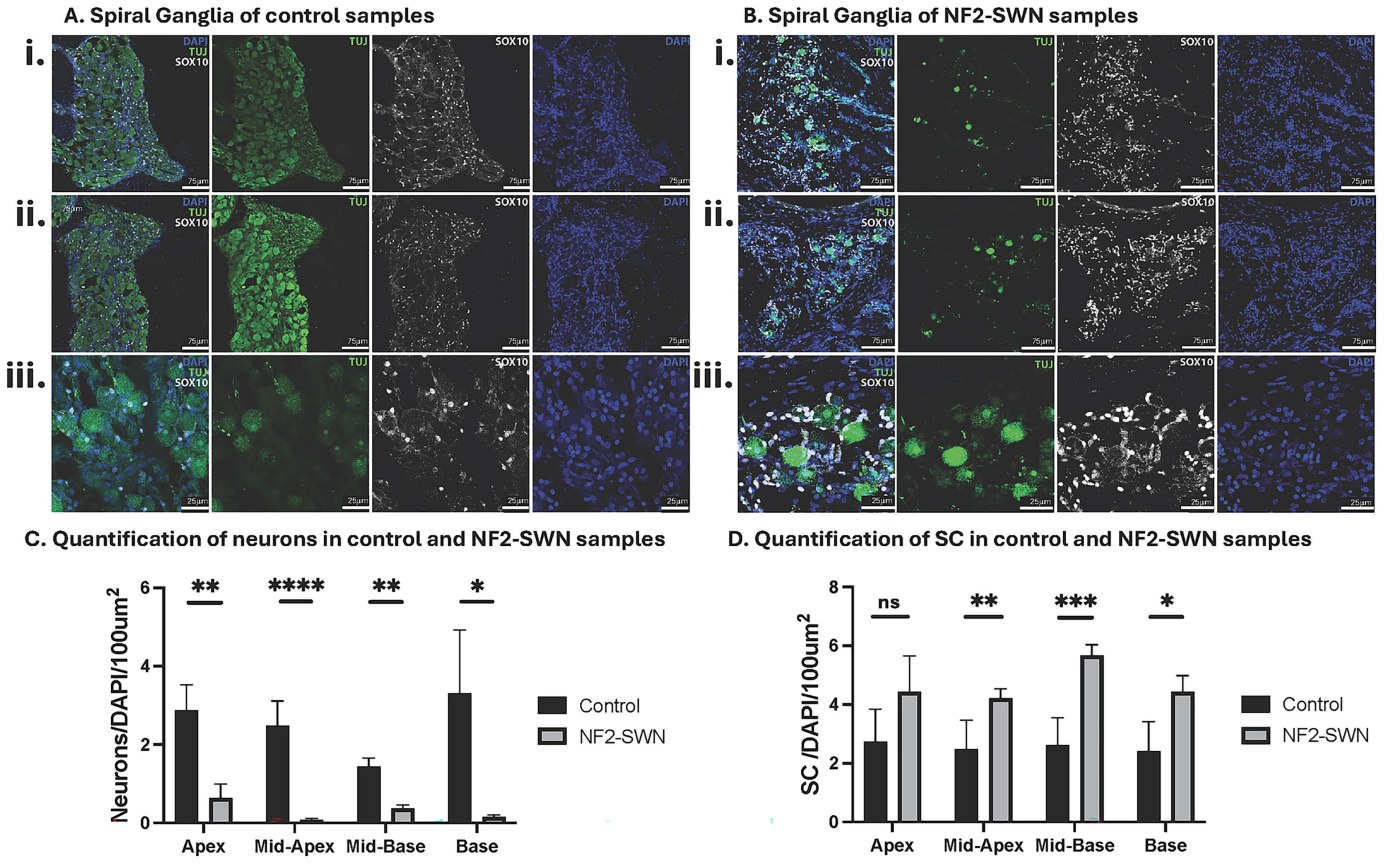
Characterization and quantification of neurons and Schwann cells in control and NF2-SWN samples. **(A,B)** Control and NF2-SWN spiral ganglia, respectively, in a mid-modiolar section. Staining: Neurons, TUJ, green; Schwann cells, SOX10+, greyscale; DAPI nuclear counterstain, blue. **(i)** Middle turn Rosenthal’s canal at 20X magnification. **(ii)** Apical turn Rosenthal’s canal at 20X magnification. **(iii)** Basal turn Rosenthal’s canal at 63X magnification. **(C)** Quantification demonstrating significantly decreased neurons in all cochlear turns in NF2-SWN as compared to controls. **(D)** Quantification of Schwann cells (SC) with significantly increased cells in middle and basal cochlear turns in NF2-SWN as compared to controls. (ns represents *p* ≥ 0.05, * represents *p* < 0.05; ** represents *p* < 0.01, *** represents *p* < 0.001, **** represents *p* < 0.0001).

**Figure 3:**
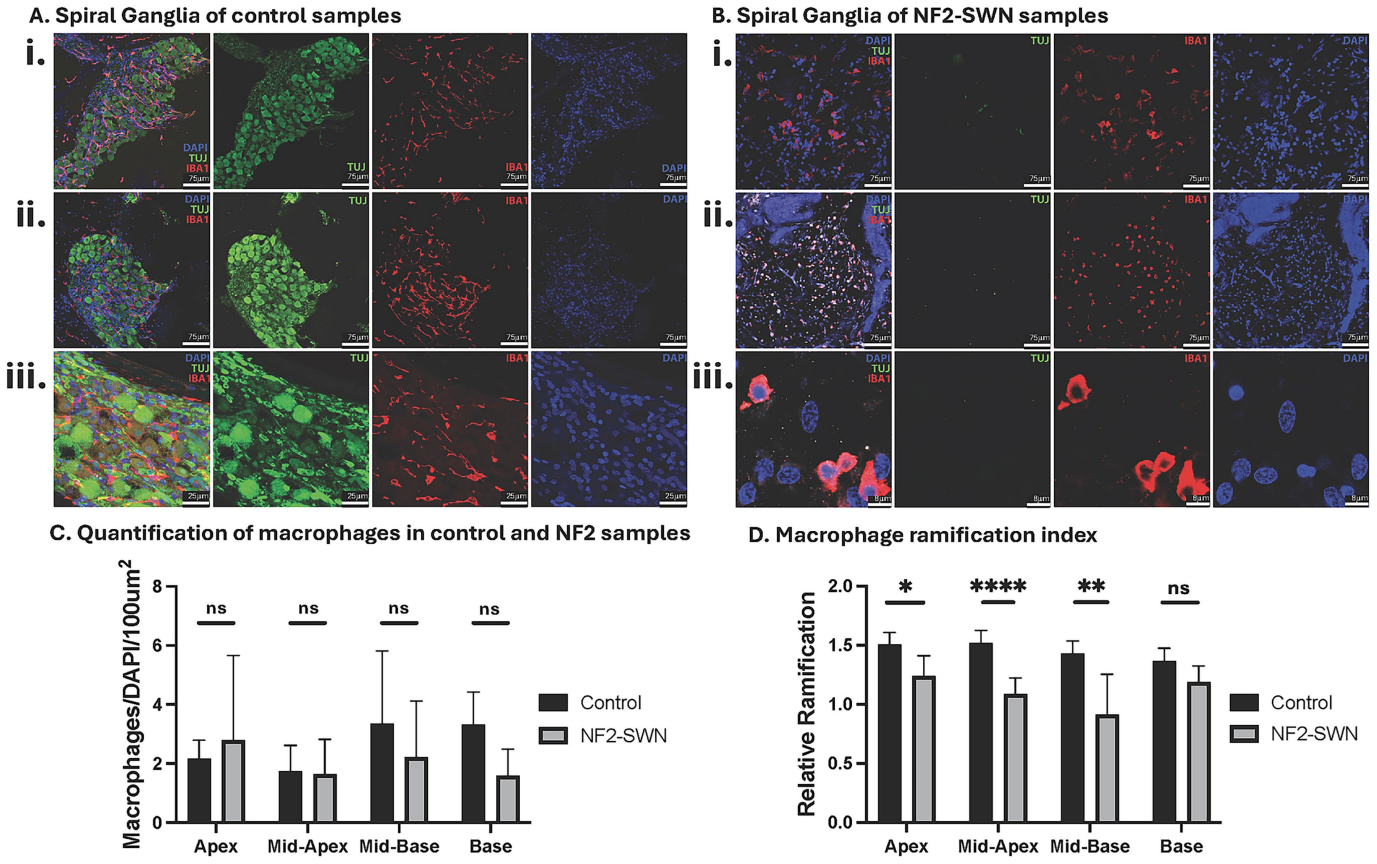
Characterization and quantification of macrophages and ramification indices in normal hearing control and NF2-SWN ears. **(A,B)** Control and NF2-SWN spiral ganglia, respectively, in a mid-modiolar section. Staining: Neurons, TUJ, green; Macrophages, IBA1+, greyscale; DAPI nuclear counterstain, blue. **(i)** Middle turn Rosenthal’s canal at 20X magnification. **(ii)** Apical turn Rosenthal’s canal at 20X magnification. **(iii)** Middle turn Rosenthal’s canal at 189X magnification. **(C)** Quantification of macrophages with no significant difference in any cochlear turns in NF2-SWN as compared to controls. **(D)** Comparison of macrophage ramification index with significantly lower ramification indices in NF2-SWN as compared to controls in apical and middle turns. (ns represents *p* ≥ 0.05, * represents *p* < 0.05; ** represents *p* < 0.01, **** represents *p* < 0.001).

Neurons were identified by positive co-staining of cytoplasmic TUJ, both centrally in the soma and peripherally in the axonal projections, and nuclear counterstain DAPI (Figures 2A,B, 3A,B). Neurons were significantly reduced in NF2-SWN samples as compared to controls in the cochlear apex (control = 2.884 ± 1.426 (*N* = 5), NF2-SWN = 0.632 ± 0.889 (*N* = 6); *p* < 0.01), mid-apex (control = 2.484 ± 1.536 (*N* = 6), NF2-SWN = 0.078 ± 0.115 (*N* = 13), *p* < 0.0001), mid-base (control = 1.435 ± 0.446 (*N* = 4), NF2-SWN = 0.366 ± 0.367 (*N* = 14), *p* < 0.01) and base (control = 3.311 ± 3.218 (*N* = 4), NF2-SWN = 0.152 ± 0.148 (*N* = 8), *p* < 0.05) (Figure 2C).

Schwann cells, identified by positive co-staining of SOX10 and DAPI, were located interstitially between neurons of Rosenthal’s canal, and along axonal projections (Figures 2A,B). They were significantly increased in the mid-apex (control = 2.487 ± 0.979 (*N* = 9), NF2-SWN = 4.209 ± 1.569 (*N* = 24), *p* < 0.01), mid-base (control = 2.608 ± 0.945 (*N* = 7), NF2-SWN = 5.677 ± 1.839 (*N* = 26), *p* < 0.001) and basal (control = 2.415 ± 1.004 (*N* = 7), NF2-SWN = 4.437 ± 2.191 (*N* = 16), *p* < 0.05) regions of NF2-SWN as compared to controls (Figure 2D).

Macrophages were identified as interstitial cells in Rosenthal’s canal and on axonal projections of SGNs with IBA1 + and DAPI co-staining (Figures 3A,B). Macrophages had no statistically significant differences in cellular quantification between NF2-SWN and control cochleae (Figure 3C). However, further qualitative investigation of cell morphology revealed conformational differences in these cells between NF2-SWN and control cochleae. Morphologically, macrophages were present in both a ramified morphology, reflecting a quiescent state, or an ameboid morphology, reflecting an activated state. Ramification index demonstrated lower values in NF2-SWN samples as compared to controls. This reduction reflects a higher proportion of activated macrophages observed in three of the four cochlear regions examined: the cochlear apex (control = 1.507 ± 0.050 (*N* = 4), NF2-SWN = 1.240 ± 0.051 (*N* = 11), *p* < 0.05), mid-apex (control = 1.517 ± 0.040 (*N* = 7), NF2-SWN = 1.089 ± 0.045 (*N* = 9), *p* < 0.0001) and mid-base (control = 1.431 ± 0.047 (*N* = 5), NF2-SWN = 0.914 ± 0.108 (*N* = 10), *p* < 0.01) (Figure 3D).

**Figure 4:**
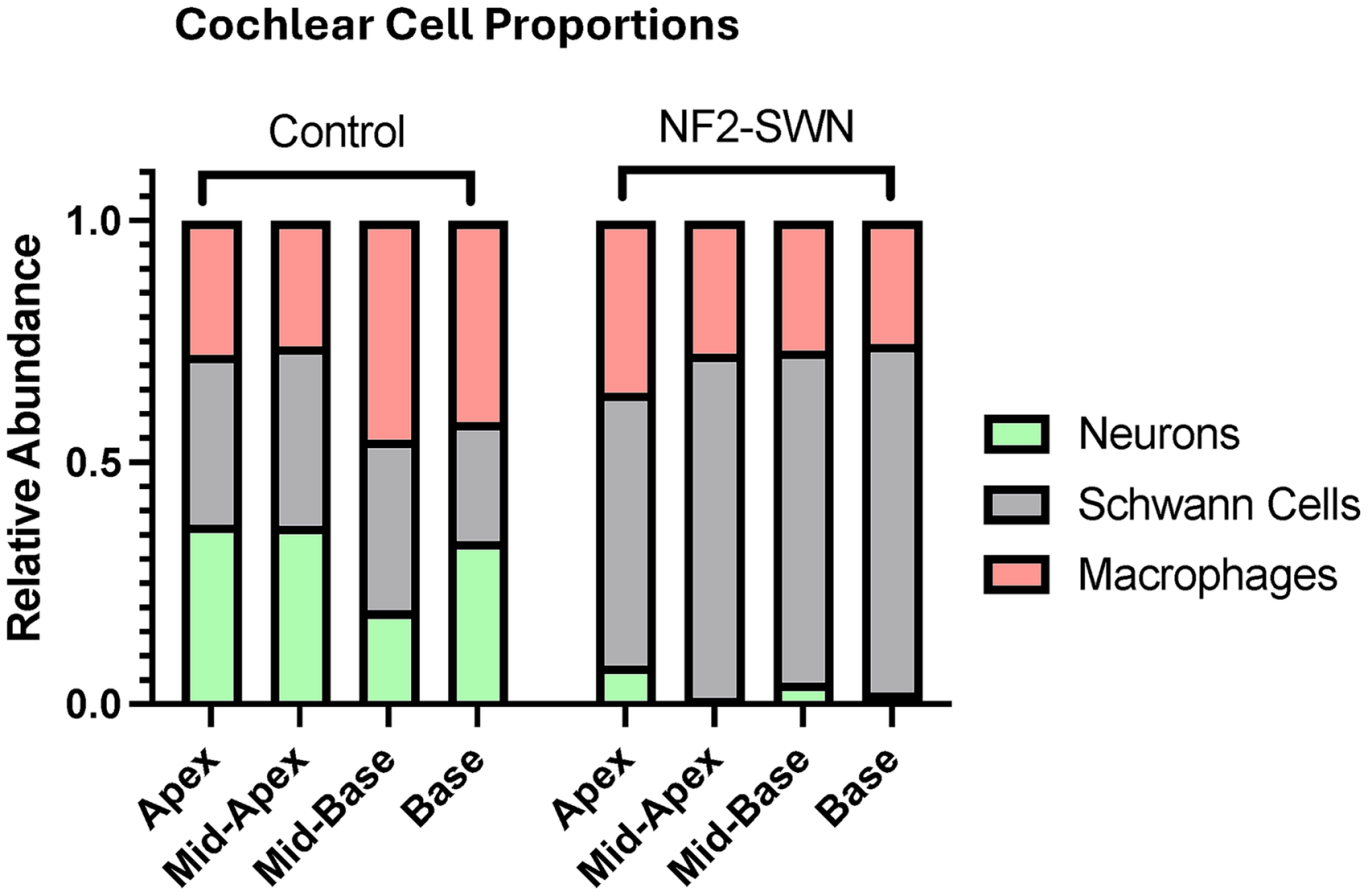
Relative abundance of neurons, Schwann cells and macrophages in control and NF2-SWN cochleae. Neurons were found to have a significantly lower proportion in NF2-SWN cochlea versus control, while Schwann cells had a significantly higher proportion. Macrophages maintained a near stable proportion in both disease and control states.

Overall, there was a significant change in the inner ear cell populations within the spiral ganglia of affected cochleae. There was a demonstrated decrease in the neuron population and increase in the Schwann cell population in NF2-SWN samples. Quantitively, cochlear macrophages were unchanged; however, a significant proportion demonstrated morphology of activated macrophages in NF2-SWN samples compared to controls (Figures 3Aiii, Biii). Controls demonstrated either relatively similar proportions of all 3 cell types or slight macrophage predominance, NF2-SWN samples were predominated by Schwann cells alongside significant reduction in neurons; the proportion of macrophages remained stable (Figure 4).

## 4 Discussion

The etiology of SNHL in patients with vestibular schwannomas, particularly in the context of NF2-SWN, remains incompletely understood. Despite the tumor typically originating from the vestibular nerve, the most common symptom of NF2-SWN is hearing loss, present in over 95% of patients (13, 14). While classical theories attributed this to mechanical compression by the tumor, emerging evidence suggests that complex, multifactorial interactions between different cell types and the cochlear and tumor environment may play a significant role in VS-associated SNHL.

Schwann cells, which proliferate in response to neuronal injury, are known to express developmental genes and facilitate axonal repair (46, 47). However, excessive Schwann cell proliferation or myelinopathy after loss of the *NF2* gene may lead to persistent neuronal stress, which may disrupt cochlear homeostasis, thereby contributing to the progressive deterioration of cochlear function and amplifying hearing loss (47–49). Furthermore, as Schwann cells continue to proliferate in response to neuronal damage, they may inadvertently exacerbate the damage by creating an environment that fosters further neuronal degeneration rather than promoting healing (47–49). Electrophysiological assessment using auditory brainstem responses in both animal models of NF2-SWN and NF2-SWN patients demonstrate a pattern of demyelination and disrupted saltatory conduction, which has been historically attributed to retrocochlear pathology (31, 33, 35, 36, 50, 51). However, mouse models of transient myelinopathy have irreversible damage at the level of the heminode (31, 35). We posit that our findings of significant Schwann cell increase in the setting of neuronal damage at the level of the cochlea similarly suggest hearing loss may be due to Schwann cell dysregulation as opposed to, or at least in addition to, retrocochlear pathology.

Macrophages serve diverse roles in the peripheral nervous system, including maintenance of homeostasis, immune surveillance, and tissue repair (40). However, persistent activation and the release of pro-inflammatory cytokines and reactive oxygen species can amplify inflammation and exacerbate neuronal damage (40). This inflammatory milieu may disrupt Schwann cell function, compounding neuronal degeneration and perpetuating the cycle of damage (25, 26, 48, 52, 53). Macrophages have also been implicated in having a regenerative role after damage in the cochlea (54). In this study, IBA1 + macrophage populations in both control and NF2-SWN temporal bone samples were comparable, yet the proportion of activated (ameboid) macrophages compared to quiescent (ramified) macrophages was significantly increased in NF2-SWN cochlea (Figures 3Aiii,Biii). This transition from quiescence to activation may reflect the presence of pro-inflammatory mediators; however, the exact mechanism of this change remains unclear. Previous studies suggest that the immune activation in NF2-SWN cochleae may be driven by release of inflammatory mediators by activated tumor-associated macrophages, including tumor necrosis factor-alpha (TNF-*α*), interleukins (IL-1β, IL-6), and reactive oxygen species, which may exacerbate neuronal damage (20, 25, 26, 48, 52, 53). This inflammatory state, if present in the cochlea, may also impair the ability of Schwann cells to support myelination or axonal regeneration (31, 52, 53). Moreover, chronic insults to tissue primes resident macrophages to become activated, which can further the cycle of inner ear damage (55).

Histological examination of vestibular schwannomas demonstrates predominance of a pattern of hypocellularity with increased immune cell infiltration, which further suggests an inflammatory microenvironment conducive to tumor progression (23, 25, 26, 28, 42, 43, 56). However, considering concurrent neuronal loss, uncontrolled Schwann cell proliferation and lack of temporal matching of SNHL and tumor growth clinically, we postulate our findings of macrophagic activation in NF2-SWN may be part of a coordinated inflammatory response within the cochlea due to aberrant Schwann cell function independent of tumor effects, and potentially contributing to pathogenesis of hearing loss.

The progressive hearing loss in NF2-SWN patients may be attributed to multiple converging mechanisms. Previous studies investigating peripheral neuropathies after loss of *NF2* gene expression demonstrate important roles for the merlin protein, including neuronal growth and regeneration (57–59). Moreover, its loss is implicated in both tumorigenesis and dysfunction of Schwann cell-neuron interaction (59). Similarly, the loss of merlin expression may cause dysfunction of Schwann cells and neurons, leading to SNHL. The loss of myelination or aberrant myelination can further impair SGN function and exacerbate hearing loss, in part potentially occurring due to Schwann cells entering a mitotic state (60). Schwann cell proliferation alone is known to recruit macrophages and other immune cells (31). Aberrant macrophage activation and subsequent inflammatory cascades within the cochlea likely contribute to neurotoxicity, further accelerating neuronal loss.

Uncertainty remains in the identification of primary therapeutic targets for treatment of hearing loss in NF2-SWN. Future research should focus on dissecting these interconnected processes, using longitudinal animal models to unravel the sequence and timing of these events, to determine whether interventions aimed at controlling immune activation or modulating Schwann cell behavior could effectively interrupt the cycle of damage.

This study is limited by its cross-sectional design, which precludes the ability to establish causality or temporal relationships between immune activation, neuronal loss, and tumor progression. The small sample size further limits the generalizability of our findings and hinders the ability to account for interindividual variability in disease progression and immune responses. Moreover, there was a limited availability to correlate immunohistochemical findings in individuals with audiometric changes, as audiologic data was limited in availability and lacked temporal consistency across individuals. Despite standardization of sample preparation by the same institution and exclusion of samples with significant artifact, variability in tissue extraction post-mortem times, processing and fixation may lead to cell population or autolytic changes that may act as confounders to our findings. Larger cohorts with robust clinical and histopathological data will be essential to validate these findings and refine our understanding of cochlear pathology in NF2-SWN.

In conclusion, this study highlights the potential role of immune activation and glial dysregulation in the pathogenesis of SNHL in *NF2*-associated vestibular schwannomas. These findings provide further evidence for the multifactorial nature of SNHL in NF2-SWN, involving neural loss, disrupted conduction, and neuroinflammation. By shifting the focus from tumor associated mechanisms to intracochlear pathology, these findings pave the way for novel therapeutic strategies aimed at preserving cochlear integrity and improving auditory outcomes in patients with NF2-SWN.

## Data Availability

The raw data supporting the conclusions of this article will be made available by the authors, without undue reservation.
